# Emotion recognition in early Parkinson’s disease patients undergoing deep brain stimulation or dopaminergic therapy: a comparison to healthy participants

**DOI:** 10.3389/fnagi.2014.00349

**Published:** 2015-01-21

**Authors:** Lindsey G. McIntosh, Sishir Mannava, Corrie R. Camalier, Bradley S. Folley, Aaron Albritton, Peter E. Konrad, David Charles, Sohee Park, Joseph S. Neimat

**Affiliations:** ^1^Department of Neurological Surgery, Vanderbilt University Medical CenterNashville, TN, USA; ^2^Department of Psychology, Vanderbilt UniversityNashville, TN, USA; ^3^Norton Neuroscience InstituteLouisville, KY, USA; ^4^Department of Neurology, Vanderbilt University Medical CenterNashville, TN, USA

**Keywords:** emotion recognition, early-stage Parkinson’s disease, healthy aging, DBS, levodopa, dopamine

## Abstract

Parkinson’s disease (PD) is traditionally regarded as a neurodegenerative movement disorder, however, nigrostriatal dopaminergic degeneration is also thought to disrupt non-motor loops connecting basal ganglia to areas in frontal cortex involved in cognition and emotion processing. PD patients are impaired on tests of emotion recognition, but it is difficult to disentangle this deficit from the more general cognitive dysfunction that frequently accompanies disease progression. Testing for emotion recognition deficits early in the disease course, prior to cognitive decline, better assesses the sensitivity of these non-motor corticobasal ganglia-thalamocortical loops involved in emotion processing to early degenerative change in basal ganglia circuits. In addition, contrasting this with a group of healthy aging individuals demonstrates changes in emotion processing specific to the degeneration of basal ganglia circuitry in PD. Early PD patients (EPD) were recruited from a randomized clinical trial testing the safety and tolerability of deep brain stimulation (DBS) of the subthalamic nucleus (STN-DBS) in early-staged PD. EPD patients were previously randomized to receive optimal drug therapy only (ODT), or drug therapy plus STN-DBS (ODT + DBS). Matched healthy elderly controls (HEC) and young controls (HYC) also participated in this study. Participants completed two control tasks and three emotion recognition tests that varied in stimulus domain. EPD patients were impaired on all emotion recognition tasks compared to HEC. Neither therapy type (ODT or ODT + DBS) nor therapy state (ON/OFF) altered emotion recognition performance in this study. Finally, HEC were impaired on vocal emotion recognition relative to HYC, suggesting a decline related to healthy aging. This study supports the existence of impaired emotion recognition early in the PD course, implicating an early disruption of fronto-striatal loops mediating emotional function.

## Introduction

While Parkinson’s disease (PD) is traditionally regarded as a neurodegenerative motor disorder, there is growing evidence for significant cognitive and emotional impairment, particularly as the disease progresses (for review see Owen, 2004). Degeneration of the substantia nigra pars compacta and the subsequent loss of dopaminergic input to the rest of the basal ganglia affects cortex through several corticobasal ganglia-thalamocortical circuits (Alexander et al., 1986). These structurally and functionally segregated loops connecting the basal ganglia to cortex are described as having motor, association (cognitive), or limbic functions (Alexander and Crutcher, 1990; Middleton and Strick, 2002; Le Jeune et al., 2008). Disruption of associative and limbic loops is thought to be one factor contributing to the accompanying cognitive decline, including poor executive functioning and memory, as well as impaired emotion recognition ability in PD (Owen, 2004).

The ability to recognize and identify emotional cues in others is a crucial component of human social interaction. Deficits in emotion recognition have been tied to poor social competence, interpersonal functioning, and communication, as well as a reduced quality of life (Ruffman et al., 2008). In regard to PD, as disease advancement leads to increased dependance on caregivers, intact emotion recognition is imperative for maintaining these intimate and essential relationships.

There is a growing literature evidencing impaired facial and vocal emotion recognition in PD and other diseases of the basal ganglia, including progressive supranuclear palsy (Gray and Tickle-Degnen, 2010; Péron et al., 2012; Pontieri et al., 2012). With respect to PD, neuroimaging work has found this impairment to be associated with bilateral orbitofrontal cortex (OFC) gray matter loss in PD (Ibarretxe-Bilbao et al., 2009). Importantly, this emotion recognition deficit is separable from an underlying visuoperceptual and cognitive dysfunction (Gray and Tickle-Degnen, 2010; Herrera et al., 2011). Deficits in vocal emotion recognition (i.e., prosody), in contrast, may not be completely separable from underlying deficits in working memory and executive function (Gray and Tickle-Degnen, 2010). While there is support for this remaining deficit for emotion recognition after factoring out dementia or other gross cognitive impairments in PD, few studies have investigated emotion recognition ability earlier in the disease progression.

Patients in the early stage of disease progression (Early PD, or EPD) are less likely to have developed cognitive impairment or dementia than their later-staged PD counterparts, as cognitive impairment is positively correlated with neuropathological staging in PD (Braak et al., 2005). A recent study documented that 70% of EPD patients showed normal cognitive performance (Elgh et al., 2009) and what deficits they do have tend to be mild (Lees and Smith, 1983; Owen, 2004). In contrast, prevalence of dementia in PD patients surviving past 90 years of age is over 80% (Buter et al., 2008). It is therefore more likely that performance of EPD patients on tasks of emotion recognition reflects the specific integrity of these emotion-related processes. The literature on emotion recognition in EPD is mixed, with several studies finding impairment similar to that of later disease stages (Sprengelmeyer et al., 2003; Dujardin et al., 2004a; Ibarretxe-Bilbao et al., 2009; Poletti et al., 2013) and others finding it generally intact (Pell and Leonard, 2005; Péron et al., 2009; Roca et al., 2010). Likely contributing to these differential findings is variation in treatment type and medication status across PD samples studied.

Two effective and common therapies for the motor symptoms of PD are dopaminergic medication and deep brain stimulation (DBS) of the subthalamic nucleus (STN). STN-DBS surgery was first reserved for advanced PD, but now is increasingly considered for those earlier in the disease progression (deSouza et al., 2013; Schuepbach et al., 2013; Charles et al., 2014) as it allows for the reduction of dopaminergic medication (Vingerhoets et al., 2002; Charles et al., 2004). Although both DBS and dopaminergic medication therapies are effective in controling motor symptoms of PD, their effects on emotion are not well understood.

A recent meta-analysis reports that the effect size for PD emotion recognition impairment to be large regardless of medication state (Gray and Tickle-Degnen, 2010). Two studies testing affect recognition in early PD samples also found no difference in task performance between medicated and unmedicated patients (Péron et al., 2009; Roca et al., 2010). However, many studies have observed either evidence for beneficial and deleterious effects of levodopa on emotion recognition in PD. Divergent findings may be explained by understanding the specific brain regions supporting the particular emotion task at hand. For instance, Cools et al. (2001, 2002, 2003) hypothesize that medication is likely to lead to enhanced performance (relative to “off” medication) for tasks involving DLPFC, where DA loss is significant. However, for tasks engaging OFC or other more ventral areas of PFC that are relatively unaffected by DA loss, medication may impair performance through an overdose action.

The literature is similarly inconclusive on the effects of STN-DBS on emotion recognition. Several studies have found no effect of STN stimulation on emotion processing (Schneider et al., 2003; Péron et al., 2010b; Brück et al., 2011; Albuquerque et al., 2014). However, others have found specific deficits in the recognition of fear (Biseul et al., 2005; Drapier et al., 2008; Péron et al., 2010a), sadness (Dujardin et al., 2004b; Drapier et al., 2008; Péron et al., 2010a), as well as anger and disgust (Dujardin et al., 2004b) post-STN-DBS surgery compared to pre-surgery. Mondillon et al. (2012) discusses possible reasons for such divergent findings on the effects of medication and stimulation on emotion recognition, and hypothesizes that STN-amygdala modulation may correct the levodopa overdose on OFC. Therefore the best emotion recognition performance may result from the combination of both levodopa and STN-DBS therapies. Additionally, methodological differences likely account for some of the variability in these findings. Interpreting results from studies comparing pre and post DBS surgery is difficult because there is often change in medication dose as well as the effect of invasive nature of the surgical procedure. Therefore, to understand the effects of stimulation itself, direct comparison of post-surgery performance with STN stimulation turned “on” and “off” is most appropriate.

The aim of this study is to investigate the ability of PD patients early in the disease course to recognize and identify emotion. By capitalizing on an ongoing prospective study examining the safety and tolerability of STN-DBS surgery in early-stage PD patients, we sought to expand our understanding of the development of neuropsychological sequelae of PD and possible effects of therapy. First, we tested emotion recognition performance across three stimulus domains (face, voice, face + voice), thereby increasing the generalizability of our findings to real life emotion perception. Second, we contrasted the effects of two kinds of therapy on emotion processing. Specifically, we capitalized on the randomized nature of the larger study to compare EPD patients who were randomly assigned to receive optimal drug therapy (ODT) to those randomly assigned to receive drug therapy plus STN-DBS (ODT + DBS). The washout nature of the larger study additionally allowed a within-subject approach of on vs. off either therapy. Third, we utilized two healthy control groups in order to separate emotion recognition impairments due to PD pathology from impairments arising from typical aging.

## Methods

### Participants

Demographic information is summarized in Table 1. Sixteen EPD patients were recruited from the Vanderbilt Clinic of Neurological Surgery from an ongoing pilot clinical trial testing the safety and efficacy of DBS therapy in early-stage PD at Vanderbilt University Medical Center (NCT00282152, IRB 040797, FDA investigational device exemption G050016) (Charles et al., 2014). Seven of these patients were recruited from a group of patients randomized to receive optimal drug therapy only (ODT group), while nine patients were recruited from a group randomized to receive STN-DBS surgery in addition to drug therapy (ODT + DBS group). Prior to completing our tasks, ODT + DBS patients were previously implanted with bilateral electrodes in the dorsolateral part of the STN using methods identical to those used in later-stage patients (Kahn et al., 2012; Camalier et al., 2014). At the time of testing, DBS stimulation amplitude averaged 1.70 V (±0.31) in left electrode, and 1.64 V (±0.25) in right electrode. All ODT + DBS patients had settings of 130 Hz and 60 μs bilaterally. Consistent with study criteria (Charles et al., 2014), all EPD patients were between the ages of 50 and 75, had a clinical diagnosis of PD, had not passed Hoehn and Yahr stage II of the disease (Hoehn and Yahr, 1967), had been treated with levodopa for more than 6 months but less than 4 years, and had not developed on/off motor fluctuations (for full inclusion criteria see Charles et al., 2014). Twenty-three healthy elderly control participants (HEC) who were matched to the EPD patients on age (*t*_(37)_ = −0.11, *p* = 0.91), sex (*χ*^2^ = 2.60, *p* = 0.11), estimated premorbid IQ (*t*_(37)_ = 1.1, *p* = 0.27), and years of education (*t*_(37)_ = 0.22, *p* = 0.83), were recruited from Nashville area. HEC had higher current IQ than EPD patients (*t*_(37)_ = 2.27, *p* = 0.03), but mean current IQ for EPD patients was still above average. In addition, 21 healthy young control participants (HYC) were recruited from the Vanderbilt University student subject pool. HYC and HEC were matched on estimated premorbid IQ (*t*_(34.00)_ = 0.70, *p* = 0.49) and sex (*χ*^2^ = 0.82, *p* = 0.37). HEC had more years of education than HYC (*t*_(31.67)_ = −2.91, *p* = 0.006), who were still in school.

**Table 1:** **Participant clinical and demographic information and performance on control tasks**.

	Healthy adults		Early Parkinson’s disease patients	
	HYC	HEC	ODT	ODT + DBS
**N**	21	23	7	9
**Sex**	12 F, 9 M	10 F, 13 M	2 F, 5 M	1 F, 8 M
**Age (years)**	20.00 ± 1.87	61.96 ± 7.76	62.29 ± 9.09	62.22 ± 7.97
**Education (years)**	13.24 ± 1.30	15.13 ± 2.80	15.86 ± 2.73	14.22 ± 2.49
**WTAR IQ**	110.25 ± 5.89	112.26 ± 11.94	108.29 ± 7.18	108.44 ± 9.81
**WASI IQ**	–	119.30 ± 11.04	111.86 ± 12.58	108.44 ± 16.76
**LEDD (mg)**	–	–	551.1 ± 197.2	348.7 ± 240.3
**UPDRS ON^*a*^**	–	–	21.43 ± 9.88	26.13 ± 14.43
**UPDRS OFF^*b*^**	–	–	37.57 ± 8.24	36.75 ± 16.99
**BFRT^*c*^**	48.62 ± 2.85	48.18 ± 3.88	42.57 ± 4.16	46.71 ± 3.73
**DTT^*d*^**	94.80 ± 6.22	91.54 ± 9.79	82.42 ± 16.89	89.74 ± 12.16

*Mean ± standard deviation (SD) reported where appropriate. ^a^UPDRS motor scores in the ON state (ODT: on medication; ODT+ DBS: on medication and on stimulation) and ^b^OFF state (ODT: off medication; ODT+ DBS: off medication and off stimulation). ^c^BFRT has a maximum score of 54. ^d^DTT performance presented as percent accuracy ± SD. EPD patients, DTT scores from ON therapy state are reported*.

All procedures were in accordance with the ethical standards of Vanderbilt University Institutional Review Board and with the Helsinki Declaration of 1975. Written informed consent was obtained from all participants after they were given a complete description of the study. HEC were paid and HYC received course credit for their participation.

### Neuropsychological testing

Pre-morbid IQ (e.g., estimated IQ before onset of disease) was estimated for all participants using the Wechsler Test of Adult Reading (WTAR; Wechsler, 2001). EPD patients and HEC also completed the Wechsler Abbreviated Scale of Intelligence (WASI; Wechsler, 1999) in order to obtain an estimated current IQ. The difference between estimated premorbid and current IQ was used to screen for evidence of early dementia (if WTAR > WASI by more than 1 standard deviation (15 index points) patient would be excluded). Consistent with the early-stage nature of the disease in this group, no EPD or HEC participants were excluded from the present study using these criteria. All testing took place at the Vanderbilt Neurosurgery Clinic or Vanderbilt Clinical Research Center.

### Procedure

EPD patients underwent 8-day inpatient evaluations at baseline optimal therapy with a 24-h period of washout from drug therapy and stimulation (see Charles et al., 2014 for full methods description). EPD patients were tested first in the ON therapy condition on day 1. In the ON condition, both groups of EPD patients were receiving optimized doses of medication, ODT + DBS patients were also receiving optimum STN stimulation as set by the clinical trial. After day 1, EPD patients began a medication washout period. In this study, OFF therapy testing occurred at least 24-h into the washout period (mean = 2.5 days, sd = 0.73). In the OFF condition, all therapy was discontinued (i.e., all patients had discontinued medications and ODT + DBS patients had also discontinued stimulation). Due to fixed study design of the larger clinical trial, testing order of therapeutic state was not counterbalanced and all patients were tested first in the ON therapy condition, and second in the OFF therapy condition. Potential effects due to practice are addressed in Section Discussion. In order to avoid interference with larger clinical study, we attempted to minimize testing time for the current study. As such, the entire testing battery was not completed for some patients. To aid in interpretation of results, the number of participants for which data was available is reported by task. Levodopa equivalent daily dose (LEDD) at study enrollment was calculated using the formula from Tomlinson et al. (2010). Average LEDD for ODT and ODT + DBS patients is reported in Table 1. Four patients (2 ODT, 2 ODT + DBS) were taking levodopa only, five patients were taking dopamine agonists only (1 ODT, 4 ODT + DBS) and seven patients were taking a combination of levodopa and dopamine agonists (4 ODT, 3 ODT + DBS). HEC and HYC participants completed all testing in one session, approximately 90 min. Unlike the EPD patients, healthy control participants completed all tasks only once. HYC completed the WTAR, but not WASI, in order to reduce testing time, as dementia was not a concern for this population.

### Testing battery

The battery included three tasks assessing emotion recognition ability across three stimulus domains: Montreal Affective Voices Task (MAV, voice; Belin et al., 2008), the Baron-Cohen Reading the Mind in the Eyes Task (RMET, face; Baron-Cohen et al., 2001), and the Awareness of Social Inference Test (TASIT, face + voice; McDonald et al., 2003, 2006). The testing battery also included two control tasks, the Benton Facial Recognition Test (BFRT; Benton, 1983) and the Distorted Tunes Test (DTT; Drayna et al., 2001).

The MAV is a validated set of 90 nonverbal affect bursts corresponding to the emotions of anger, disgust, fear, pain, sadness, surprise, happiness, and pleasure, and neutral. The voices belong to five male and five female actors, each actor vocalizing the nine emotions (Belin et al., 2008). After hearing each stimulus, participants chose between the nine options on the screen. MAV has been previously used to assess prosodic emotion recognition in PD (Eitan et al., 2013), progressive supranuclear palsy (Ghosh et al., 2012) as well as schizophrenia (Alba-Ferrara et al., 2013) and major depressive disorder (Naranjo et al., 2011). Neuroimaging results show poor MAV performance associated with gray matter loss in right inferior frontal and left middle frontal gyri (Ghosh et al., 2012). Electrophysiological evidence also points to involvement of ventral area of the right STN during this task (Eitan et al., 2013).

The RMET is a set of 36 black and white images of the eye region of faces, designed to assess the affective component of Theory of Mind (Tom; Baron-Cohen et al., 2001). Each stimulus is presented with four adjective answer choices. Participants are instructed to choose the word that best describes what the person in the image is thinking or feeling. Answer choices extend beyond the Ekman (1972) six emotions generally considered to be universal, with complex options such as *fantasizing, contemplative, playful*, and* amused*. While not a classic emotion recognition task, RMET has been previously used to assess affective processes in PD (Péron et al., 2009, 2010c; Bodden et al., 2010; Roca et al., 2010; Poletti et al., 2011; Tsuruya et al., 2011), autism (Baron-Cohen et al., 2001), and schizophrenia (meta-analysis: Bora et al., 2009). Posterior superior temporal sulcus and inferior frontal gyrus are among the cortical areas thought to support RMET performance (Adams et al., 2010; Moor et al., 2012).

The TASIT consists of videotaped vignettes of everyday social interactions designed to assess various components of social perception. TASIT has been used in clinical populations including traumatic brain injury (TBI; McDonald and Flanagan, 2004; McDonald and Saunders, 2005; Williams and Wood, 2010), frontotemporal dementia (Kipps et al., 2009), chronic depression (Ridout et al., 2007), and schizophrenia (Kern et al., 2009; Roberts and Penn, 2009). In the current study, we used a TASIT subtest, the Emotion Evaluation Test (EET), to assess the recognition of spontaneous emotional expression (happy, surprised, sad, anxious, angry, disgusted, and neutral). Participants were shown 28 audiovisual vignettes, with a seven forced-choice response following the end of each trial. TASIT consists of forms A and B, which were counterbalanced by testing session in the EPD groups, and by participant in the HEC and HYC groups. Impaired emotion recognition performance on TASIT has been found in patients with nonspecific TBI (McDonald et al., 2003; McDonald and Flanagan, 2004) as well as those who have undergone anterior cingulotomies for depression (Ridout et al., 2007).

The BFRT is a standardized measure with 27 items used to assess the ability to discriminate and match different faces. It is often used to screen for lower-level visuoperceptual impairments, particularly in studies assessing facial affect recognition (Hayes et al., 2009; Yamada et al., 2009; Hot et al., 2013). Poor performance on BFRT has been linked to lesions to right posterior-inferior parietal and right ventral occipitotemporal cortex (Tranel et al., 2009). Due to the small sample size we did not use BFRT as a tool to screen and exclude subjects, but we included the measure in the testing battery in order to quantify facial processing ability across the groups. For EPD groups, BFRT was administered once, during the ON therapy state for each patient.

The DTT is an auditory processing task with 26 items that requires participants to judge whether simple popular melodies contain notes with an incorrect pitch (Drayna et al., 2001; Braun et al., 2008). Used in other clinical populations as a tool to assess sensory deficits (Yang et al., 2012), poor performance on DTT is associated with deficits in processing speech sounds (Jones et al., 2009). Paired with MAV, DTT allows us to determine whether impairment in vocal emotion recognition is attributable to lower-level auditory processing impairment.

All tasks, with the exception of BFRT, were presented on a computer screen. In order to avoid confounds due to alterations in motor state, participants provided verbal responses, which were recorded by experimenter, for all tasks apart from RMET. Due to verbal response format and instructed emphasis on accuracy, reaction time was not collected or measured for any task.

### Data analysis

Mean percent accuracy was calculated for DTT, TASIT, RMET, and MAV. BFRT was scored out of a possible total long form score of 54. Statistical tests were performed using SPSS. Due to the small number of participants in ODT and ODT + DBS groups, for a subset of the analyses these groups were combined to form one EPD group for comparison with HEC. HEC performance and that of EPD patients in the ON condition (first testing condition) were compared to investigate the effect of PD pathology on emotion recognition. To elucidate effects of PD therapy type (ODT/ODT + DBS) as well as state (ON/OFF), ODT and ODT + DBS emotion recognition performances were compared using repeated-measures ANOVA design. HEC and HYC performances were compared to isolate effects of typical aging on these tasks. For comparisons between EPD and HEC, results are reported with and without controling for group differences in current IQ (WASI) and BFRT scores.

## Results

Clinical and demographic information for all participants is provided in Table 1.

### Performance on control tasks: Benton and DTT

HEC had higher BFRT scores than ODT (*t*_(27)_ = 3.28, *p* = 0.003) but not DBS (*t*_(27)_ = 0.88, *p* = 0.17) patients. Although they made more errors than HEC, on average ODT patients performed in the “normal” range on BFRT (score of ≥41; Benton, 1983). HEC also had higher DTT scores than ODT, though this did not reach statistical significance (*t*_(25)_ = 1.75, *p* = 0.09). HEC and ODT + DBS patients had very similar DTT scores (*t*_(27)_ = 0.42, *p* = 0.68). Means ± standard deviation (SD) for BFRT and DTT performance are reported in Table 1.

### Effect of PD therapy type and state

For each emotion recognition task, a 2 (treatment type: ODT/ ODT + DBS) × 2 (treatment state: ON/OFF) repeated-measures ANOVA was conducted on the mean percent accuracy. There was no significant effect of treatment type (*p* > 0.4 for all tasks) or treatment state (*p* > 0.5 for all tasks) on emotion recognition tasks. There was, however, a significant effect of therapy state on DTT performance (*F*_(1,14)_ = 4.82, *p* = 0.045). This was the result of ODT performing worse on DTT during OFF state, as ODT + DBS patients showed no ON/OFF differences in performance. For the DTT interaction between therapy type and state, there was a moderate trend toward significance (*F*_(1,14)_ = 3.41, *p* = 0.09). Means and SD for these analyses are reported in Table 2.

**Table 2:** **Task performance in the ON and OFF therapy state for ODT and ODT + DBS patients**.

Task	Therapy state	ODT		ODT + DBS	
		*N*	Mean ± SD	*N*	Mean ± SD
**DTT^*a*^**	*ON*	7	82.42 ± 16.89	9	89.74 ± 12.16
	*OFF*	7	77.47 ± 13.22	9	89.32 ± 13.43
**TASIT**	*ON*	6	72.02 ± 7.30^*b*^	9	71.43 ± 13.60
	*OFF*	4	74.11 ± 10.26	9	71.03 ± 15.09
**RMET**	*ON*	6	67.13 ± 6.90	8	63.22 ± 12.93^*c*^
	*OFF*	6	70.37 ± 5.46	7	65.62 ± 21.18
**MAV**	*ON*	7	62.22 ± 8.19	9	59.38 ± 9.38
	*OFF*	7	63.49 ± 6.92	9	59.63 ± 10.57

*Means shown are % accuracy ± SD. N = Number of subjects per condition and task. ^a^p < 0.05 for effect of therapy state. For within-subjects analyses comparing ON/OFF performance, patients with data available from only one testing session were not included. For ^b^TASIT, two ODT patients were excluded from within-subject analysis for this reason and the adjusted mean ± SD for the remaining four subjects in the ON therapy state is 69.64 ± 6.84, and for ^c^RMET, one ODT + DBS patient was excluded and the adjusted mean ± SD for the remaining seven subjects in the ON therapy state is 66.25 ± 10.46*.

### Effect of healthy aging on emotion recognition

Means ± SD and *t*-values for emotion recognition tests are reported in Table 3. HEC and HYC performed similarly overall on TASIT (*t*_(37)_ = 0.65, *p* = 0.52). However, with respect to specific emotion recognition performance, HEC performed significantly better than HYC at identifying disgusting videos (*t*_(23.18)_ = −2.13, *p* = 0.04). The opposite was true for neutral videos (*t*_(37)_ = 2.63, *p* = 0.01). There was no effect of age on RMET performance (*t*_(36)_ = 0.35, *p* = 0.73). There is a significant effect of age on overall MAV performance (*t*_(29.65)_ = 4.57, *p* < 0.001), due to HEC performing worse than HYC. This age-related impairment was evident in specific emotion conditions including anger (*t*_(34)_ = 2.98, *p* = 0.005), fear (*t*_(34)_ = 4.79, *p* < 0.001), happy (*t*_(20.00)_ = 4.18, *p* < 0.001), neutral (*t*_(22.79)_ = 4.26, *p* < 0.001), and pain (*t*_(34)_ = 2.42, *p* = 0.02).

**Table 3:** **Performance for HYC, HEC, and EPD patients on emotion recognition tests TASIT, MAV, and RMET**.

		HYC vs. HEC	HYC	HEC	EPD	HEC vs. EPD
		*t*				*t*
**TASIT**	*N*		19	20	15
	*Overall*	−0.65	86.47 ± 7.95	84.64 ± 9.56	71.67 ± 11.17	3.70**
	*Neutral*	−2.63*	92.11 ± 11.94	81.25 ± 3.75	56.67 ± 19.97	4.32**
	*Disgusted*	2.13*	81.58 ± 26.14	95.00 ± 10.26	81.67 ± 17.59	2.62*
	*Anxious*	−1.05	89.47 ± 15.17	83.75 ± 18.63	66.67 ± 29.48	2.10*
	*Angry*	−1.66	88.16 ± 15.29	80.00 ± 15.39	83.33 ± 24.40	−0.50
	*Sad*	0.77	80.26 ± 17.83	85.00 ± 17.01	73.33 ± 20.00	1.86
	*Happy*	−0.37	80.26 ± 17.83	77.50 ± 27.98	60.00 ± 24.64	1.93
	*Surprised*	−0.89	93.42 ± 11.31	90.00 ± 12.57	80.00 ± 16.90	2.01
**MAV**	*N*		15	21	16
	*Overall*	−4.57**	78.37 ± 4.56	66.98 ± 10.06	60.62 ± 8.71	2.02
	*Neutral*	−4.26**	98.67 ± 5.16	76.67 ± 22.88	68.75 ± 25.53	0.99
	*Disgusted*	−0.76	82.00 ± 11.46	77.62 ± 19.98	74.38 ± 11.53	0.58
	*Afraid*	−4.79**	66.00 ± 11.83	41.43 ± 17.11	37.50 ± 18.80	0.66
	*Angry*	−2.98**	54.67 ± 19.95	36.67 ± 16.23	23.13 ± 15.37	2.57*
	*Sad*	−1.72	96.00 ± 6.32	90.00 ± 14.14	88.75 ± 10.25	0.30
	*Pain*	−2.43*	62.67 ± 16.24	47.62 ± 19.72	43.75 ± 18.93	0.60
	*Happy*	−3.65**	100.00 ± 0.00	92.86 ± 7.84	89.38 ± 10.63	1.15
	*Pleasure*	−0.39	76.00 ± 23.84	72.86 ± 23.48	73.75 ± 13.60	−0.14
	*Surprised*	−0.36	69.33 ± 17.51	67.14 ± 18.75	46.25 ± 20.29	3.24**
**RMET**	*N*		19	19	14
	*Overall*	0.12	77.98 ± 11.05	76.68 ± 11.69	64.90 ± 10.60	2.98**

Means shown are % accuracy ± SD. *p < 0.05, **p < 0.01.

### Effect of EPD pathology on emotion recognition

Because we found no effect of PD therapy type on emotion recognition performance between the ODT and ODT + DBS groups, we combined these two groups into one (EPD) so that we would be better powered to detect group differences on tasks with HEC. Means ± SD and *t*-values for HEC and EPD performance on emotion recognition tests are reported in Table 3.

TASIT (emotion recognition from faces + voices): *T*-tests found that EPD patients performed worse than HEC on TASIT overall (*t*_(33)_ = 3.70, *p* = 0.001). Specific affect impairment was found for disgusted, anxious, and neutral. Statistical trends for specific affect impairments were found for sad (*p* = 0.07), happy (*p* = 0.06), and surprised (*p* = 0.05). Group effect remained significant after controling for current IQ (*F*_(1,32)_ = 7.76, *p* = 0.009). BFRT performance was found to be unrelated to TASIT accuracy (*F*_(1,30)_ = 1.02, *p* = 0.32).

RMET (emotion recognition from faces): EPD patients also performed worse than HEC on RMET (*t*_(31)_ = 2.98, *p* = 0.006). This effect remained significant at trend level (*F*_(1,32)_ = 3.73, *p* = 0.06) after controling for current IQ. BFRT performance was unrelated to RMET accuracy (*F*_(1,27)_ = 0.04, *p* = 0.84).

MAV (emotion recognition from voices): *T*-tests showed EPD patients performed worse than HEC on MAV (*t*_(35)_ = 2.02, *p* = 0.05). The groups also diverged in performance for angry (*t*_(35)_ = 2.57, *p* = 0.02), and surprised (*t*_(35)_ = 3.24, *p* = 0.003) conditions. After controling for current IQ, EPD and HEC performed similarly overall on MAV (*F*_(1,34)_ = 0.679, *p* = 0.42).

## Discussion

We found that EPD patients are impaired on emotion recognition across three types of stimuli relative to matched healthy controls. Impairment on facial emotion recognition tasks (RMET, TASIT) was not accounted for by underlying differences in ability to process basic facial features. EPD patients’ vocal emotion recognition impairment (MAV) was less pronounced, though specific deficits in processing of angry and surprised voice stimuli were found. Similar performance on DTT between EPD patients and healthy aged matched controls indicate this impairment is not accounted for by differences in low-level pitch perception. These group effects were differentially affected by controling for current IQ (WASI), though this potential confound is tempered by the fact that the EPD patients in this study had an average IQ of 108 and thus had typical cognitive functioning.

Our finding of facial emotion recognition impairment in EPD replicates that of a meta-analysis showing this impairment in later stages of PD (Gray and Tickle-Degnen, 2010). Evidence of brain regions supporting the affect recognition tasks in the present study partially overlap with known projections from basal ganglia to frontal cortex via the associative and limbic loops. Thus, our findings suggest these loops may be dysfunctional early in the disease process. Our finding of impaired emotion recognition from eyes (RMET) does conflict with two studies (Péron et al., 2009; Roca et al., 2010), which have found intact RMET performance in early PD. One important methodological difference between these former studies and the current study, which may account for divergent results, is the other studies’ use of abbreviated version of the RMET, with half the number of stimuli used in the current study. Most recently, Poletti et al. (2013), using the same full 36-item RMET used in the current study, also found impaired performance in EPD patients relative to matched healthy controls. While the EPD patients included in this study were also impaired on emotion recognition from voice stimuli (MAV), the effect was weaker than those for face stimuli (RMET, TASIT). This is in contrast to a meta-analysis that found larger effect size for PD emotion recognition impairment for vocal stimuli compared to that of faces (Gray and Tickle-Degnen, 2010). It is possible that prosody recognition deteriorates over time as PD progresses, but that remains to be tested longitudinally.

Our results indicate that dopaminergic medication and STN-DBS do not differentially affect emotion recognition performance in EPD. Likely playing a part in many studies that contrast these two therapies is underlying patient clinical and neuropsychological differences between the treatment groups. For example, some PD patients are not willing to undergo surgery for treatment, or surgery is contraindicated due to other medical concerns. Furthermore, existing neurocognitive deficits such as impairment in semantic fluency, or problems with impulse control, affect the decision to provide surgical intervention and certainly the surgical target for DBS (e.g., STN v. globus pallidus interna (GPi)). An important feature of the current study is that EPD patients were assigned to their treatment groups through a completely randomized process. Perhaps, then, these patient characteristics that often clinically influence a therapy choice are not a factor for this study and our results reflect this.

We do not see an effect of STN stimulation on emotion recognition in this sample. This finding is in contrast to previous findings that STN-DBS surgery leads to deficits in emotion recognition, particularly for negative emotions (Dujardin et al., 2004b; Drapier et al., 2008; Péron et al., 2010a). However, methodological differences between previous studies and ours are of note. In this study, patients receiving STN stimulation (ODT + DBS) were of early PD progression, not past Hoehn and Yahr stage II. Additionally, this study isolates the effect of STN-DBS stimulation from that of DBS surgery, which may confer its own effects on emotion recognition processes (Brück et al., 2011) as well as executive function (Okun et al., 2009). There is extremely limited evidence for the effect of STN-DBS stimulation in early PD, and for this reason it is very difficult to compare our finding to the published literature on STN stimulation effects. In comparison to published studies examining STN stimulation in advanced PD patients, stimulation levels in this study (ODT + DBS group) were relatively low; under 2 V. It is possible that this low setting is efficacious for controling motor symptoms but does not exert measurable extra-motor side effects. Another possibility is potential disruptive effects of stimulation, exhibited in the other studies, is only evidenced as disease progression itself produces greater deficits in emotional processing. In view of the concerns for cognitive change associated with STN-DBS in advanced PD, this study seems to indicate that STN-DBS, when applied early in the therapy, does not worsen the pre-existing decline in emotional processing already present early in the disease.

A similar lack of ON/OFF effect for emotion recognition was found on dopaminergic medication therapy in the ODT group. The literature on the effect of dopaminergic medication on emotion processing in PD (most of which has been conducted in moderate to severe PD populations) is mixed. It is possible that early in the disease progression, levodopa or dopamine agonists do little to affect emotion recognition processes. Within the EPD literature, our finding that emotion recognition is unaffected by medication status is in accordance with other studies (Péron et al., 2009; Roca et al., 2010). Separate from the emotion recognition battery used in this study, the DTT did reveal an effect of therapy state, potentially an effect of medication. DTT was employed in this study as a perceptual control task, not expected to be affected by treatment state. This finding certainly requires further investigation.

Our finding of EPD impairment on tests of emotion recognition is consistent with previous work documenting altered structure and function of OFC and amygdala in early disease stages. Ouchi et al. (1999) observed reduced density of dopamine transporter binding sites in OFC and amygdala in levodopa naïve, early PD patients. Consistent with progression of Lewy-body pathology to neocortex (Braak et al., 2003), OFC (Ibarretxe-Bilbao et al., 2009; Tinaz et al., 2011) and amygdala (Ibarretxe-Bilbao et al., 2009) volumes are decreased by Hoehn and Yahr stage II. OFC volume is strongly correlated with emotion recognition performance in EPD patients (Ibarretxe-Bilbao et al., 2009). However, functional MRI study has suggested that abnormal amygdala activation during perception of emotional faces is present in EPD prior to manifestation of behavioral impairment in emotion recognition (Tessitore et al., 2002).

It is important to consider how neuropsychological assessments for emotion recognition or discrimination abilities may be included in neuropsychological assessments for PD. Early detection of an emotion recognition deficit could direct the patient to interventions, for instance, social cognition training, to remediate the impairment before it worsens. While not yet studied in PD, such interventions are found to be effective in improving social cognition in disorders such as schizophrenia (Horan et al., 2008; Roberts and Penn, 2009) and autism (Turner-Brown et al., 2008). Emotion recognition tests employed in research could be easily adapted for neuropsychological assessment, assuming sufficient normative data exists. For example, Ekman and Friesen’s (1975) classical facial affect recognition test is now a subtest in the Social Cognition and Emotional Battery (SEA; Bertoux et al., 2012; Funkiewiez et al., 2012), used for early diagnosis of the behavioral variant of frontotemporal dementia (bvFTD), where aberrant social behavior is often the presenting symptom. The RMET (Baron-Cohen et al., 2001) is already widely used in research for assessing ToM in different clinical populations. While there is some normative data available (Baron-Cohen et al., 2001), we know little about how healthy older adults perform on this task, preventing present adoption of the task in neuropsychological assessment of the elderly. Although not greatly impaired in this EPD sample, deficits in vocal emotion recognition are more striking than for facial emotion recognition in later stages of PD (Gray and Tickle-Degnen, 2010). To our knowledge, no neuropsychological testing includes vocal emotion recognition tests. However, the Montreal Affective Voices Battery (Belin et al., 2008) could be standardized and eventually included alongside tests of facial affect recognition in neuropsychological assessments.

Finally, it is still relatively unknown how deficits on tests of emotion recognition translate into patients’ every day social behaviors, so future research should endeavor to link test performance with a real-life measure of social functioning. Additionally, the development of more ecologically valid measurements of social cognition, such as gaze behavior, is warranted (Sturm et al., 2011; see also Kumfor and Piguet, 2013).

### Effect of aging on emotion recognition

By including two healthy control groups in this study, we were able to capture effects of typical aging on emotion recognition. Our finding of a robust HEC impairment on MAV, relative to HYC, indicates that decoding of emotional prosody deteriorates with age. This is in accordance with previous studies investigating the affect of aging (Ruffman et al., 2008; Mill et al., 2009), reporting deficits in vocal emotion processing in older adults. Importantly, there was no difference between HYC and HEC in performance on DTT, our auditory control task. This suggests that the finding cannot be attributed to age-related hearing loss, which is also in line with previous research (Orbelo et al., 2005; Mitchell, 2007). However, other studies (Mill et al., 2009; Ruffman et al., 2008, 2009) have also reported that facial affect recognition also deteriorates with age, particularly that of negative emotions. This HEC sample performed similarly to HYC on our facial emotion recognition tasks, TASIT and RMET, with the exception of neutral and revolted videos in the TASIT. However, most previous studies investigating facial affect recognition use static face stimuli, different from that used in the current study. TASIT requires the integration of contextual verbal and non-verbal information from social interaction. RMET provides only the eye region of faces, so perhaps it is easier focusing on only this region, though studies have observed aging related impairment on this task as well (see Kemp et al., 2012 for review). Additionally, the answer choices for RMET are dissimilar to those used in typical affect recognition tasks, as it was originally designed to assess affective ToM. It is possible that these methodological differences could account for the differences between our study and previous studies investigating the effects of healthy aging.

### Limitations

It is important to note certain limitations to our study. Due to study constraints, we were unable to counterbalance the therapeutic state of the testing sessions in the EPD patients; ON testing was always first and OFF testing always second. This limitation is shared by many studies investigating the effect of STN-DBS on emotion recognition, as a pre vs. post surgery approach is frequently employed to investigate effects of stimulation. Future studies investigating the effect of PD therapy on emotion recognition using a similar within-subject design would ideally counterbalance testing sessions. We were unable to compare all possible therapy states for EPD-DBS group (e.g., DBS-ON/ MED-ON, DBS-ON/MED-OFF, DBS-OFF/MED-ON, DBS-OFF/MED-OFF). Our ability to compare all possible testing states was limited by concerns for EPD patients’ comfort and desire to minimize potential practice effects. Future studies should compare all possible testing states in order to completely separate stimulation from medication effects in PD-DBS patients. Additionally, as this study was initiated after the larger clinical trial was initiated, pre-surgical testing of emotion recognition was not possible. Furthermore, EPD patients were not blinded to therapy state, making placebo effects a possible confound in our results. Finally, our EPD sample is small, which may have hindered our ability to detect group differences in such a heterogeneous disease. As such, the study may have been underpowered to detect effects of therapy type and state on emotion recognition. Unfortunately, given the constraints of the larger clinical trial, a larger group of EPD patents could not be sampled. Interpretation of these results would benefit from replication in a larger sample.

## General conclusions

This study finds emotion recognition impairment in early PD. This impairment was strongest for tasks of facial affect recognition. These deficits are not accounted for by lower-level impairment in visual or auditory processing. It is interesting to find such deficits in patients who otherwise exhibited a normal cognitive profile. It suggests that alterations of the basal ganglia circuitry effects both motor and limbic domains before broader cognitive effects are observed in later PD (Braak et al., 2005). We found no difference treatment type (ODT/ODT + DBS) on emotion recognition performance in this early PD sample. The patients who participated this study were randomized to treatment groups as part of a larger study, which allowed us to test early PD patients receiving DBS stimulation; a rare population. This randomized design also avoids many confounds in patient characteristics that may otherwise have affected task performance, such as neurocognitive deficits or problems in impulse control. Nevertheless, this finding would benefit from replication in a larger sample. By utilizing two healthy control groups (young and elderly), we were able to replicate the finding that emotional prosody identification decays with age, and provide evidence that facial affect recognition remains intact in individuals in their 60 s. Our results support the existence of altered functioning in non-motor corticobasal loops early in the PD progression, which is likely associated with documented disturbances in OFC and amygdala in EPD patients. Early deficits in the ability to interpret social cues seen in this study are especially concerning as progressive motoric impairment in PD often leads to increased reliance on caregivers. Researchers and clinicians in this field should work toward the inclusion of emotion perception tests into neuropsychological assessments to aid in identifying patients who are significantly impaired in this domain. Provision of education and support surrounding these interpersonal deficits as a symptom of the disease may be warranted in an effort to preserve important relationships with loved ones. Whether these deficits could be improved through intervention is a critical area that is yet to be explored in PD.

## Author contribution

Lindsey G. McIntosh collected and analyzed data, wrote, and revised the final manuscript. Sishir Mannava collected and analyzed data, and wrote a first draft of the manuscript. Corrie R. Camalier contributed to study conception and design, analyzed data, revised and critiqued the manuscript and provided supervision. Bradley S. Folley obtained funding, contributed to study conception and design, and reviewed and critiqued the manuscript. Aaron Albritton collected data and reviewed and critiqued the manuscript. Sohee Park contributed to study conception and design and reviewed and critiqued the manuscript. Peter E. Konrad obtained funding, reviewed and critiqued manuscript, and provided supervision. David Charles obtained funding, reviewed and critiqued manuscript, and provided supervision. Joseph S. Neimat obtained funding, contributed to study conception and design, reviewed and critiqued manuscript, and provided supervision.

## Conflict of interest statement

Vanderbilt University receives income from grants or contracts with Allergan, Ipsen, Medtronic, and Merz for research or educational programs led by David Charles. David Charles receives income from Allergan, Ipsen, Medtronic, Merz, and the Alliance for Patient Access for education or consulting services. Peter Konrad receives income from Medtronic for research grants and consulting services. Joseph Neimat receives income from Medtronic and Monteris Medical Inc. for consulting services. Lindsey G. McIntosh, Sishir Mannava, Corrie R. Camalier, Bradley S. Folley, Aaron Albritton and Sohee Park have no conflicts of interest.
